# Presumed Infected Bronchogenic Cyst Presenting as Acute Pericarditis: A Case Report

**DOI:** 10.7759/cureus.106059

**Published:** 2026-03-29

**Authors:** Xante Mentens, Yanina Jansen, Karel Wallecan, Lynn Decoster

**Affiliations:** 1 Internal Medicine, University of Antwerp, Antwerp, BEL; 2 Department of Thoracic surgery, University Hospital Leuven, Leuven, BEL; 3 Department of Radiology, Turnhout General Hospital, Turnhout, BEL; 4 Department of Pulmonary Medicine, Turnhout General Hospital, Turnhout, BEL

**Keywords:** bronchogenic cyst, congenital malformation, mediastinal cyst, mediastinal mass, pericardial effusion, pericarditis, robot-assisted thoracoscopic surgery, thoracic surgery

## Abstract

Bronchogenic cysts are rare congenital anomalies arising from abnormal budding of the ventral foregut during early embryogenesis. They are typically located in the mediastinum or pulmonary parenchyma and are mostly asymptomatic. However, complications such as infection, compression, hemorrhage, or rupture may cause significant morbidity. Surgical resection is recommended in symptomatic or complicated cysts and may also be justified in asymptomatic patients to prevent future complications. We report the case of a 40-year-old male who presented with chest pain and dysphagia. A presumptive diagnosis of a mediastinal bronchogenic cyst complicated by acute pericarditis and probable secondary infection was made based on the clinical presentation and imaging findings. The patient underwent robot-assisted thoracoscopic surgery, and histopathological analysis confirmed the diagnosis of a bronchogenic cyst. This case highlights a rare but clinically significant complication of a bronchogenic cyst.

## Introduction

Bronchogenic cysts are the most common congenital cystic lesions of the mediastinum, accounting for approximately 10-15% of all primary mediastinal masses and over half of mediastinal cysts [1-3]. Despite this, they remain relatively rare, with an estimated prevalence of one in 42000 to 68000 individuals based on surgery and autopsy series [3]. These cysts arise from abnormal budding of the tracheal diverticulum from the ventral foregut and are typically located near the tracheobronchial tree or within the lung parenchyma, although atypical locations have been reported [3,4].

Histologically, bronchogenic cysts are lined by ciliated respiratory epithelium and usually contain fluid or mucus [1,3]. More than 90% of patients are asymptomatic, with most cysts identified incidentally on thoracic imaging [1]. However, when complications occur, they can present with symptoms related to mass effect, infection, hemorrhage, or rupture. In rare cases, these complications may be severe and potentially life-threatening [2,3,5].

We report the case of a 40-year-old male with an infected bronchogenic cyst complicated by acute pericarditis.

## Case presentation

A 40-year-old male presented to the emergency department with retrosternal chest pain, aggravated in the supine position and with deep inspiration. He also reported dysphagia for solid foods, generalized malaise, and diaphoresis but denied fever. His past medical history was notable for childhood asthma and a soft tissue sarcoma of the right thigh at age 14, treated with chemoradiotherapy and surgical resection. The patient was not on any regular medication at the time of presentation.

On admission, physical vital signs were within normal limits, with a blood pressure of 104/68 mmHg, heart rate of 76/min, oxygen saturation of 97% on room air, and body temperature of 36.4°C. Physical examination was unremarkable; in particular, no pericardial friction rub was noted. Laboratory investigations showed elevated inflammatory markers, including a C-reactive protein (CRP) level of 99.0 mg/L (<5.0 mg/L) and leukocytosis of 17.0 x 10^9^/L (3.9-8.8 x 10^9^/L). D-dimers and serial troponin levels were within normal limits (Table 1).

**Table 1:** Laboratory results

Parameter	Value	Normal range
C-reactive protein	99.0 mg/L	<5.0 mg/L
White blood cell count	17.0 x 10^9^/L	3.9-8.8 x 10^9^/L
Troponin	<3.2 ng/L	<34.2 ng/L

Nasopharyngeal swab testing for SARS-CoV-2, influenza A and B, and respiratory syncytial virus yielded negative results. Electrocardiography (ECG) showed a normal sinus rhythm with widespread concave ST-segment elevations appreciated throughout most limb leads (I, II, III, aVF) and several precordial leads (V3-V6).

**Figure 1:**
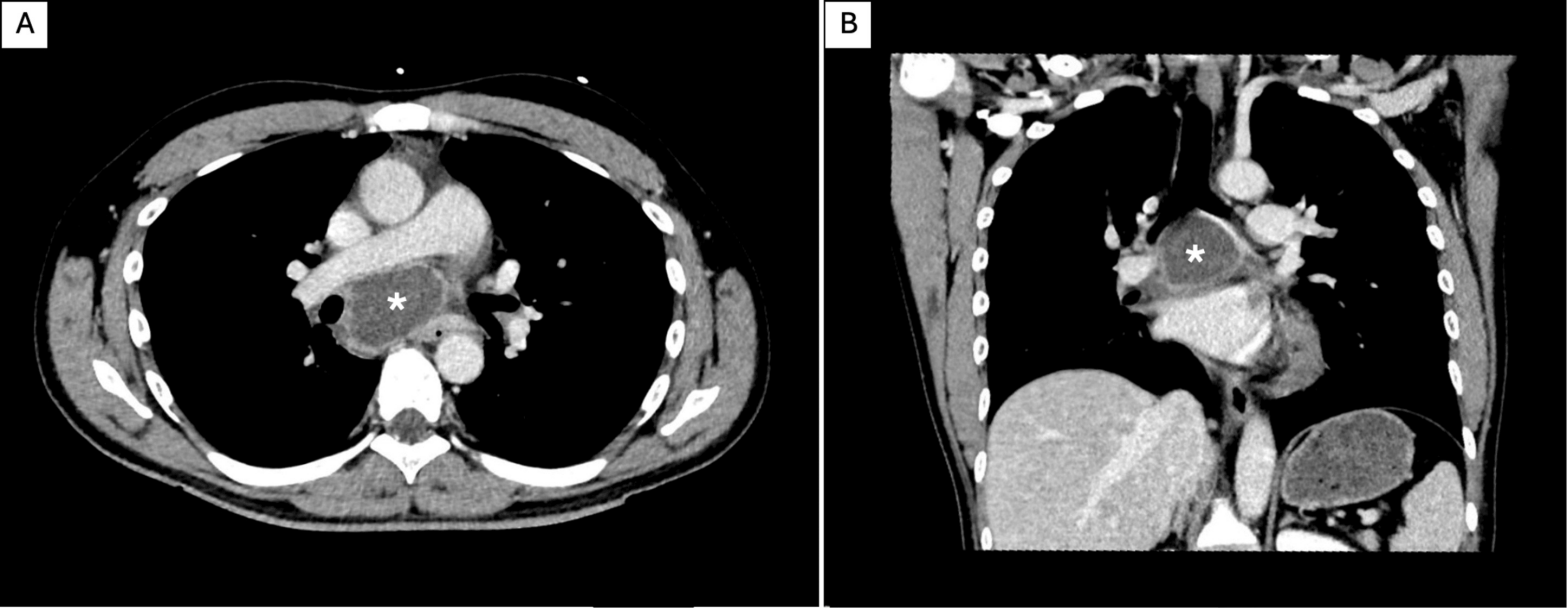
Computed tomography (CT) at presentation Contrast-enhanced CT of the chest with axial (A) and coronal (B) reformats at the level of the cyst demonstrates a rounded cystic mass measuring 5.6 cm in maximum diameter, located in the subcarinal region (white asterisk). The lesion exhibits smooth peripheral rim enhancement in venous contrast phase with a non-enhancing hypoattenuating center consistent with internal fluid content (0-20 Hounsfield Units).

Given the combination of dysphagia and chest pain, a contrast-enhanced computed tomography of the neck and thorax was performed. Although chest radiography is typically the initial imaging modality, in this case, the emergency physician proceeded directly to computed tomography (CT) due to concern for alternative acute mediastinal pathologies, including aortic dissection. In this clinical context, CT was considered the most appropriate first-line imaging modality, and a chest radiograph was therefore not obtained prior to the CT scan. CT imaging revealed a well-defined subcarinal cystic lesion measuring 3.3 × 4.6 × 5.6 cm, exhibiting smooth peripheral rim enhancement in the venous contrast phase. A small pericardial effusion and mildly enlarged mediastinal lymph nodes were also noted (Figure 1).

Transthoracic echocardiography confirmed the presence of the pericardial effusion without any signs of cardiac tamponade or other functional impairment.

**Figure 2:**
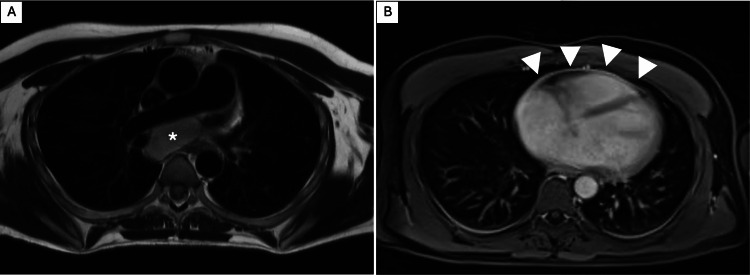
Magnetic resonance imaging (MRI) three weeks after treatment with amoxicillin-clavulanate and oral acetylsalicylic acid Axial T2-weighted image at the level of the subcarinal cyst (A) demonstrates a relatively high internal signal intensity consistent with fluid content (white asterisk), and a thin hypointense rim that enhanced after gadolinium (not shown). No internal septations or solid components are seen. Post-contrast T1-weighted VIBE (volumetric interpolated breath-hold examination) sequence with gadolinium at the level of the heart (B) reveals persistent mild pericardial thickening with associated linear enhancement (arrows), indicative of pericardial inflammation. The small pericardiac effusion has since resolved.

Based on the characteristic clinical presentation, typical electrocardiographic findings, and the presence of a pericardial effusion on CT and echocardiography, a diagnosis of acute pericarditis was made in accordance with established diagnostic criteria. Alternative diagnoses, including pneumonia with pleuritic pain, pulmonary embolism, acute coronary syndrome, and aortic dissection, were excluded based on biochemical and imaging findings. Given the anatomical proximity of the cystic lesion to the pericardium, its location, and the contrast enhancement of its wall on CT, we hypothesized that an infected bronchogenic cyst induced a reactive pericarditis. The associated mediastinal lymphadenopathy observed on CT was also considered secondary to the infectious process. The patient was treated with intravenous amoxicillin-clavulanate (1.2 g four times daily) and oral acetylsalicylic acid (1 g four times daily) with clinical and biochemical improvement. He was discharged on oral antibiotics pending further evaluation. Magnetic resonance imaging (MRI) three weeks later showed a persistent subcarinal cyst with mild residual inflammation, while the pericardial effusion had resolved (Figure 2). 

**Figure 3:**
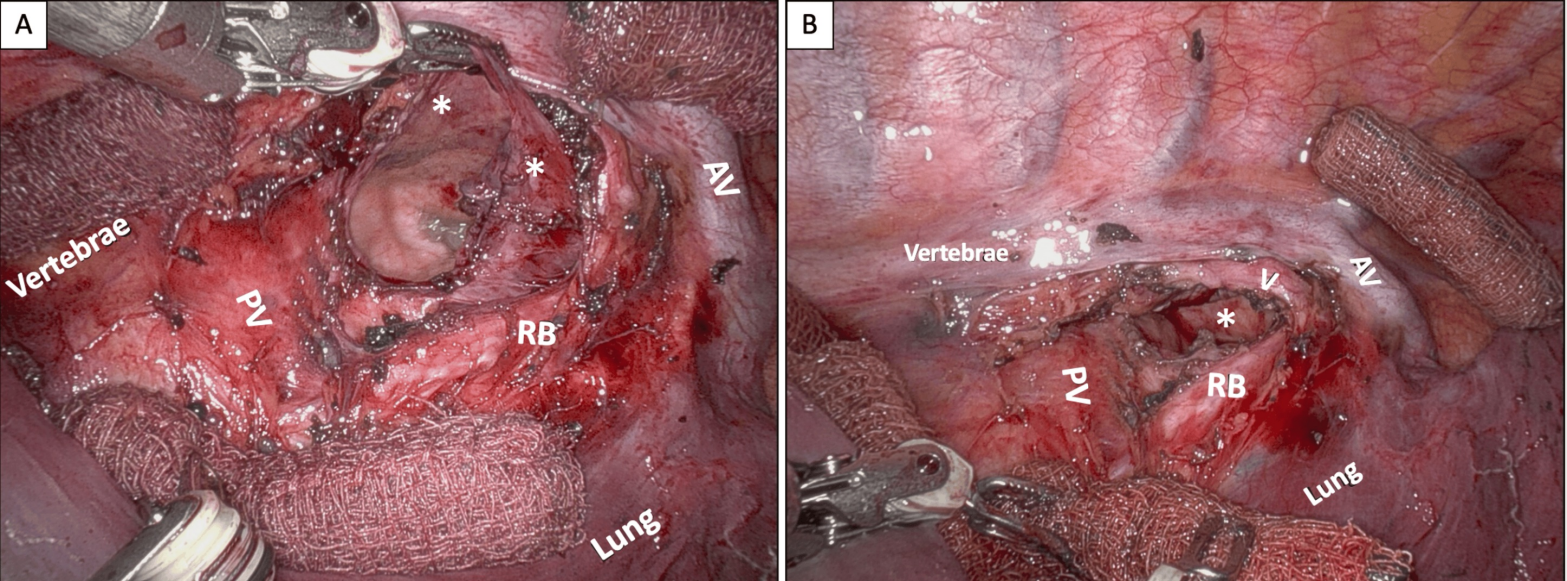
Robotic resection of the bronchogenic cyst Intraoperative view after opening the cystic wall during robotic resection (A) with greenish mucoid content visible within the bronchogenic cyst. View after marsupialization of the cyst (B) demonstrating clear anatomical exposure of the inferior pulmonary vein, right main bronchus, and contralateral pericardium. PV: inferior pulmonary vein; AV: azygos vein; V: vagal nerve; RB: right main bronchus; white asterisk in A: wall of the bronchogenic cyst; white asterisk in B: contralateral pericardium.

Given the complicated nature of the bronchogenic cyst, robot-assisted thoracoscopic surgery (RATS) was performed. The cyst was identified and dissected as extensively as possible, taking into account its close proximity to the vagal nerve, the right main bronchus, and the inferior pulmonary vein. Marsupialization was then performed by deroofing the cyst and thereby creating a wide communication between the cyst cavity and the right thoracic cavity. This approach was intended to allow any recurrent cyst fluid to drain into the pleural space, thereby reducing the likelihood of re-encapsulation. Cauterization of the cyst wall was not carried out in our patient. A green, mucoid fluid was drained from the cyst and sent for microbiological analysis (Figure 3).

**Figure 4:**
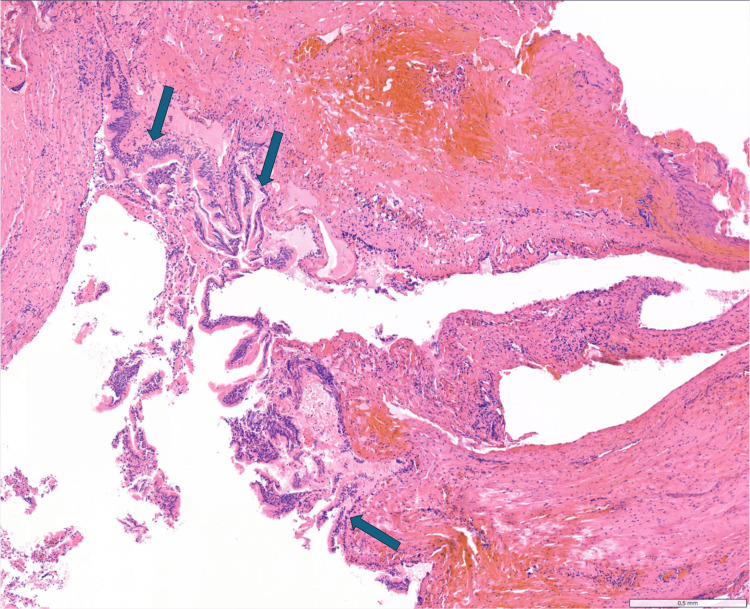
Histopathological examination Histology of resection specimen (hematoxylin-eosin, ×5), showing a mediastinal cyst lined by pseudostratified ciliated columnar epithelial cells showing no atypia (blue arrows).

Histopathological examination confirmed the diagnosis of a bronchogenic cyst, showing a cyst wall lined by a multilayered respiratory epithelium without any atypical cells (Figure 4).

The cyst wall also showed dense fibrous connective tissue, consistent with chronic inflammatory change. Microbiological cultures were negative; however, they should be interpreted with caution, as samples were obtained intraoperatively after a three-week course of antibiotic therapy. Despite the lack of microbiological confirmation, the clinical, biochemical, and intraoperative findings support an infected bronchogenic cyst. Initially, the patient had an elevated CRP that normalized with antibiotic treatment, and intraoperatively, the cyst contained greenish fluid, favoring an infectious rather than sterile inflammatory process. Together, these findings indicate that the cyst was likely infected despite negative microbiological cultures.

**Figure 5:**
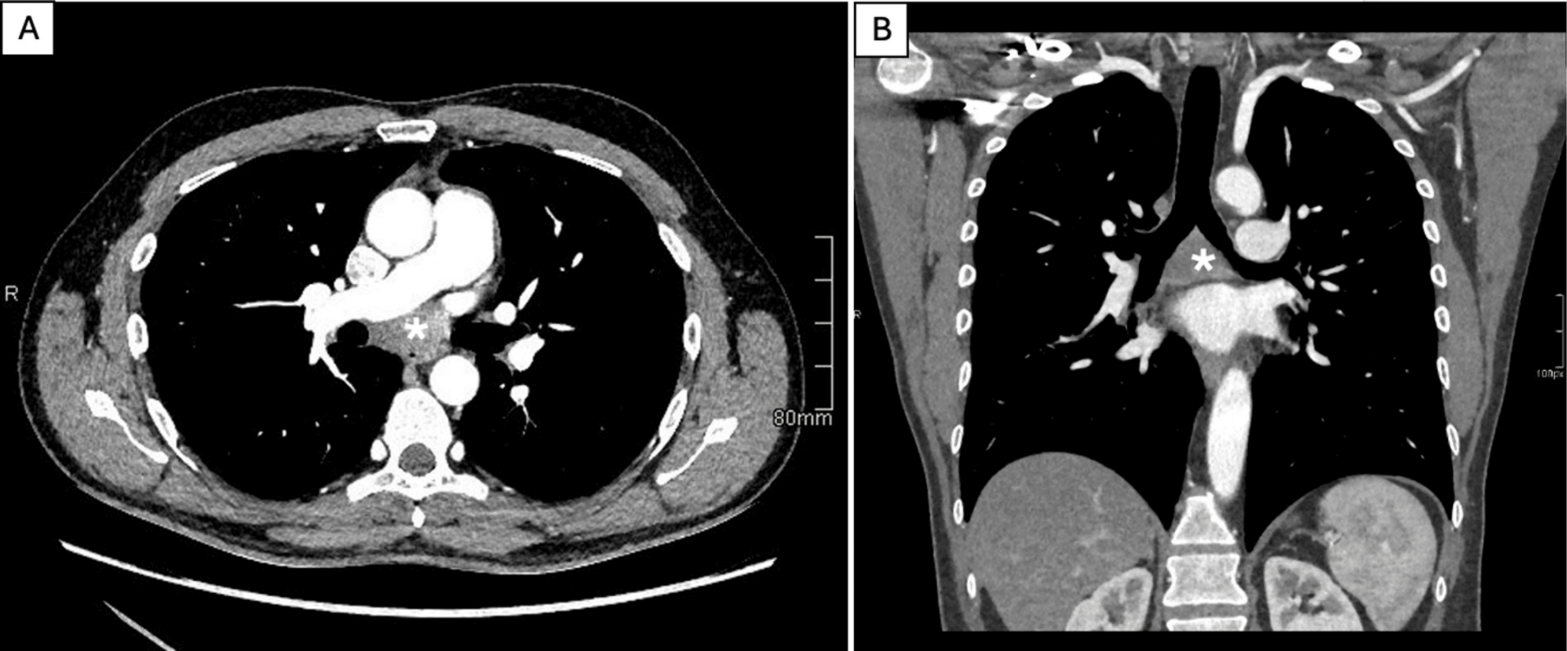
Computed tomography (CT) at the six-month follow-up Six months following excision, a persistent residual lesion of approximately 3.5 cm width (white asterisk) is noted in the subcarinal space with internal fluid contents of variable density (30-60 Hounsfield units), possibly representing a remnant of the primary bronchogenic cyst.

The postoperative course was uneventful. At the three-month follow-up, the patient reported intermittent thoracic discomfort and reduced exercise tolerance, although ECG had normalized. At six months, the patient reported complete resolution of pain and normal physical activity. CT showed a persistent subcarinal lesion with internal fluid content of variable density, possibly a remnant of the bronchogenic cyst (Figure 5). 

## Discussion

Bronchogenic cysts are rare congenital anomalies that result from abnormal budding of the tracheobronchial tree from the ventral foregut between the third and seventh week of gestation [1,3]. While they most commonly occur in the mediastinum (50-70%) adjacent to the tracheobronchial tree or within the pulmonary parenchyma (20-30%), they have also been reported in atypical locations, including the diaphragm, retroperitoneum, pericardium, thymus, abdomen, spine, cervical region, and even in the skin and subcutaneous tissue [1,3,6,7]. While bronchogenic cysts can be found in the heart, they are uncommon, representing only 1.3% of primary cardiac and pericardiac tumors [8]. Histologically, bronchogenic cysts are typically unilocular and contain clear serous fluid, mucus, proteinaceous debris, or occasionally air or blood. The cyst wall is characteristically lined by pseudostratified ciliated columnar respiratory epithelium and may contain additional airway components, such as smooth muscle fibers, mucous glands, or cartilage [1,2,6]. Acute or chronic infection, hemorrhage, or infarction may alter the cyst wall or content over time [3,5].

Although bronchogenic cysts are typically benign and slow-growing, they may cause significant complications depending on their size and anatomical location. While often asymptomatic, these cysts can become symptomatic due to infection, compression on surrounding structures, rupture, or fistulization into adjacent organs such as the airway. Clinical manifestations are generally nonspecific and may include chest pain radiating to the back or abdomen, dyspnea, palpitations, cough, dysphagia, or recurrent respiratory infections [1,3,6,8]. In the case of fistula formation, symptoms such as fever, productive cough, or hemoptysis may also be reported [3]. Compression-related complications can be severe and include pulmonary artery stenosis, superior vena cava syndrome, bronchial atresia, unilateral pulmonary edema, obstructive emphysema, pneumothorax, and pleural effusion [7,8]. Cardiac involvement may manifest as pericardial effusion or tamponade, atrial fibrillation or ventricular arrhythmias, atrioventricular block, or myocardial infarction due to coronary artery compression [6-8]. Malignant transformation is exceedingly rare but has been reported [3,7]. Occasionally, bronchogenic cysts are associated with other congenital abnormalities, such as esophageal duplication cysts and cardiac defects including atrial and ventricular septal defects, patent ductus arteriosus, tetralogy of Fallot, and pericardial agenesis [3,8-10].

Bronchogenic cysts are most commonly diagnosed in early childhood, particularly when respiratory symptoms develop as a result of infection or mass effect. However, asymptomatic lesions may remain undetected until adulthood and are frequently discovered incidentally by imaging performed for unrelated indications [3]. The differential diagnosis is broad and varies depending on the anatomic location of the cyst. Intrapulmonary cysts may mimic hydatid cysts, primary or metastatic malignancies, granulomas, hematomas, vascular malformations, pulmonary sequestration, pulmonary abscesses or infected bullae, fungal infections, or tuberculosis. Mediastinal cysts must be distinguished from vascular anomalies, hydatid cysts, malignancies, thymic or pericardial cysts, esophageal duplication cysts, lymphangiomas, cystic teratomas, lipomas, lymphomas, germ cell tumors, and reactive lymphadenopathy [1,3,7]. 

Clinical examination and routine laboratory investigations are often nonspecific (e.g., abnormal lung auscultation, tachypnea, hypoxia, a palpable mass, elevated C-reactive protein, or leukocytosis) but can be useful in identifying complications [3]. Imaging plays a central role in the diagnosis. A chest radiograph is typically the first diagnostic modality, and may reveal a solitary, round or oval mass with air or fluid content or an air-fluid level [1,3]. CT is the imaging method of choice, providing detailed information about the size, location, and anatomical relationships of the lesion [3]. Bronchogenic cysts typically appear as well-defined, homogeneous, hypodense, round or oval masses [1,6]. Classic cysts have water-like attenuation (0-20 Hounsfield units), although the presence of proteinaceous, calcific, or hemorrhagic content can increase density values and complicate differentiation from solid tumors [7]. CT is also valuable for identifying complications such as infection or compression of adjacent structures [3]. MRI offers superior delineation of complex anatomical relationships and is particularly useful for preoperative planning, although it is not routinely required [1,3]. Signal characteristics depend on the cyst’s contents; T1-weighted images may show high or low signal intensity depending on mucus, blood, or protein-rich content, whereas T2-weighted images typically show high signal intensity without contrast enhancement [3,4]. Bronchoscopy may be useful to assess airway compression, and endoscopy with endobronchial ultrasound (EBUS), which is a minimally invasive, outpatient procedure, can guide fine-needle aspiration for diagnostic or therapeutic purposes [3]. Despite the availability of high-quality imaging modalities, definitive diagnosis requires histopathological confirmation from surgically resected tissue or biopsy specimens [1,3].

Management strategies for bronchogenic cysts depend on their size and location but are primarily based on the presence of symptoms and risk of complications. Surgical resection remains the treatment of choice for symptomatic or complicated bronchogenic cysts [1]. This approach is associated with low perioperative morbidity and mortality and offers excellent long-term outcomes with low recurrence rates [3]. Surgical management of mediastinal masses has shifted from open thoracotomy to minimally invasive techniques such as video-assisted thoracic surgery (VATS) and RATS. Compared with open procedures, VATS offers reduced operation time, less blood loss, less pain and shorter hospital stay, but the technique is limited by restricted visualization and instrument flexibility [11,12]. RATS retains the advantages of VATS while providing high-definition three-dimensional imaging and improved visibility, reduced hand tremor, and enhanced precision, enabling safer and less traumatic resections of masses in the confined mediastinal space [11,12]. However, if complications occur or if visibility and dissection planes are unclear during minimally invasive approaches, there should be a low threshold to convert to open thoracotomy [12]. Complete excision is preferred, however when not feasible due to involvement of critical surrounding structures, subtotal resection must be performed with cauterization of the residual cyst wall or injection with toxic agents into the wall to reduce the risk of recurrence [1,3,4,13]. Several case reports have described the use of marsupialization when complete surgical resection was not feasible, as was the case in our patient [14,15]. Incomplete excision of the cyst is generally associated with an increased risk of recurrence; however, cases without recurrence following partial removal have also been reported [6,12,13]. Recurrence typically occurs after a prolonged interval of several years, and recurrent cysts tend to present with more pronounced symptoms and a higher rate of complications compared to primary cysts [13]. Symptomatic patients awaiting surgery may require supportive therapy with analgesics, bronchodilators, and anti-inflammatory agents for pain and dyspnea relief, or antibiotics in case of infection [3]. For patients not suitable for surgery, image-guided percutaneous drainage or endobronchial drainage via EBUS-guided transbronchial needle aspiration can provide temporary relief of symptoms; however, it is associated with a high recurrence rate and is not considered a definitive treatment [3,7,12]. 

The management of incidentally discovered, asymptomatic bronchogenic cysts remains a matter of debate [1,3,4,7]. A conservative approach with close radiological surveillance is considered reasonable in selected cases. However, clinical trials have shown that up to 45% of asymptomatic cysts eventually become symptomatic, and approximately 0.7% carry a risk of malignant transformation [1,7,16]. Therefore, elective surgical resection may be justified even in asymptomatic patients, particularly in young children and otherwise healthy adults, to prevent future symptoms or complications and to avoid possible malignant transformation [1,3,16].

## Conclusions

Bronchogenic cysts, though rare and typically benign, should be considered in the differential diagnosis of cystic thoracic lesions. Our case highlights how infection of an otherwise asymptomatic bronchogenic cyst can precipitate acute clinical manifestations such as acute pericarditis and dysphagia, which may act as the initiating event leading to its diagnosis. This emphasizes the importance of a thorough diagnostic work-up, including detailed imaging studies, as these congenital anomalies are mostly incidental findings, but can also cause severe, atypical symptoms. Management decisions should carefully weigh the risk and benefits of conservative versus surgical approaches, recognizing that even asymptomatic cysts may become complicated over time. Complete surgical resection remains the definitive treatment of choice, offering excellent long-term outcomes and low recurrence risk and preventing future complications. Awareness of this condition is crucial for timely recognition and management.
